# Protein-Lipid Interaction of Cytolytic Toxin Cyt2Aa2 on Model Lipid Bilayers of Erythrocyte Cell Membrane

**DOI:** 10.3390/toxins12040226

**Published:** 2020-04-03

**Authors:** Sudarat Tharad, Boonhiang Promdonkoy, José L. Toca-Herrera

**Affiliations:** 1 Institute for Biophysics, Department of Nanobiotechnology, University of Natural Resources and Life Sciences (BOKU), 1190 Vienna, Austria; 2 National Center for Genetic Engineering and Biotechnology, National Science and Technology Development Agency, Pathumthani 12120, Thailand; boonhiang@biotec.or.th

**Keywords:** Cyt2Aa2 toxin, protein-lipid binding, erythrocyte membrane, AFM, QCM-D

## Abstract

Cytolytic toxin (Cyt) is a toxin among *Bacillus thuringiensis* insecticidal proteins. Cyt toxin directly interacts with membrane lipids for cytolytic action. However, low hemolytic activity is desired to avoid non-specific effects in mammals. In this work, the interaction between Cyt2Aa2 toxin and model lipid bilayers mimicking the erythrocyte membrane was investigated for Cyt2Aa2 wild type (WT) and the T144A mutant, a variant with lower hemolytic activity. Quartz crystal microbalance with dissipation (QCM-D) results revealed a smaller lipid binding capacity for the T144A mutant than for the WT. In particular, the T144A mutant was unable to bind to the phosphatidylcholine lipid (POPC) bilayer. However, the addition of cholesterol (Chol) or sphingomyelin (SM) to the POPC bilayer promoted binding of the T144 mutant. Moreover, atomic force microscopy (AFM) images unveiled small aggregates of the T144A mutant on the 1:1 sphingomyelin/POPC bilayers. In contrast, the lipid binding trend for WT and T144A mutant was comparable for the 1:0.4 POPC/cholesterol and the 1:1:1 sphingomyelin/POPC/cholesterol bilayers. Furthermore, the binding of WT and T144A mutant onto erythrocyte cells was investigated. The experiments showed that the T144A mutant and the WT bind onto different areas of the erythrocyte membrane. Overall the results suggest that the T144 residue plays an important role for lipid binding.

## 1. Introduction

The most widely known bacteria as a bioinsecticidal agent is *Bacillus thuringiensis* (Bt). It is a Gram-positive rod shape bacterium originally hosted in soil. In the last few decades Bt has been used to control insect larvae especially for pest insects in the form of a bioactive agent or a transgenic plant. The active proteins, Crystal (Cry) and Cytolytic (Cyt) toxins are produced as crystalline proteins during the sporulation phase of the Bt growth cycle [1]. After toxin ingestion by insect larvae, the protein crystals are solubilized and concomitantly activated by proteases in alkaline condition of the mid gut [2,3,4]. Consequently, the toxins interrupt a cell membrane permeability of the gut cells leading to cell burst because of the osmotic pressure imbalance [5,6]. However, both toxins disrupt the cell membrane with different mechanisms. Cry toxin requires a protein receptor for the cell membrane binding whereas Cyt toxin interacts directly with the membrane lipids [7,8,9], in particular with the unsaturated phospholipids [10]. Cry toxin has been used more in crop fields to control insect larvae than Cyt toxin because of its efficiency and specificity [11]. Nevertheless, the long-term application of Cry toxin has led to insect larvae resistance [12]. Accordingly, Cyt toxin has been taken into the strategy to overcome such resistance. Thus, the Cyt toxin is able to be a receptor for the Cry toxin and these toxins can synergize their activities together [13].

Cytolytic toxin Cyt2Aa2 is produced from *Bacillus thuringiensis* subsp. *darmstadiensis* [14]. The Cyt toxin shows the cytolytic activity against a broad range of cell types, e.g., insect cells, mammalian cells [7], and bacterial cells [15]. Previous experiments suggest that the protein-lipid binding mechanism of the Cyt toxin is driven by (i) pore formation [16,17], (ii) detergent-like action [18,19], and (iii) carpet action (protein aggregate) [20]. However, the precise mechanism is still unclear and devotes further investigation. In particular, hemolytic activity has been tested to determine the cytolytic activity of Cyt2Aa2 toxin against erythrocyte cells (in relation to mammalian cells). Therefore, we have tried to obtain a variant with lower hemolytic activity by performing amino acid mutation (in order to reduce the non-specific target to mammalian cells). The effect of the amino acid point mutation of the Cyt2Aa2 molecule on the toxin activity has been reported. Previous studies have shown that the amino acids located in the helix A, helix C [21], and helixD-beta4 loop [22,23] alter the activity of the Cyt2Aa2 toxin. Particularly, we have investigated the amino acid mutation of T144A (alanine replacement of T144 residue) placed in the helixD-beta4 loop (Figure S1) because it keeps its larvicidal activity, although its hemolytic activity is reduced [24].

To elucidate the influence of the point mutation T144A on the interaction of Cyt2Aa2 protein with model lipid bilayers (which mimic the erythrocyte membrane), we have carried out binding studies with the Cyt2Aa2 wild type (WT) and the mutant Cyt2Aa2-T144A. The prepared lipid bilayers containing phospholipid (POPC), sphingomyelin (SM), and cholesterol (Chol) were mixed in various molar ratios to build the different membranes, which can be found in erythrocyte cells [25,26]. The combination of quartz crystal microbalance with dissipation (QCM-D) and atomic force microscopy (AFM) indicated that the T144A mutant had a lower binding capability than the WT, especially for the POPC bilayer. Moreover, the T144A mutant formed small aggregates on the 1:1 SM/POPC bilayer (showing a different binding trend from the Cyt2Aa2 WT). Finally, both toxins were also exposed to lysed erythrocytes cells. It was found that WT and T144A bound to different parts of the erythrocyte membrane.

## 2. Results

### 2.1. Determination of the Cyt2Aa2-Lipid Interaction with Different Model Lipid Bilayers that Mimic the Erythrocyte Membrane by QCM-D

**Figure 1:**
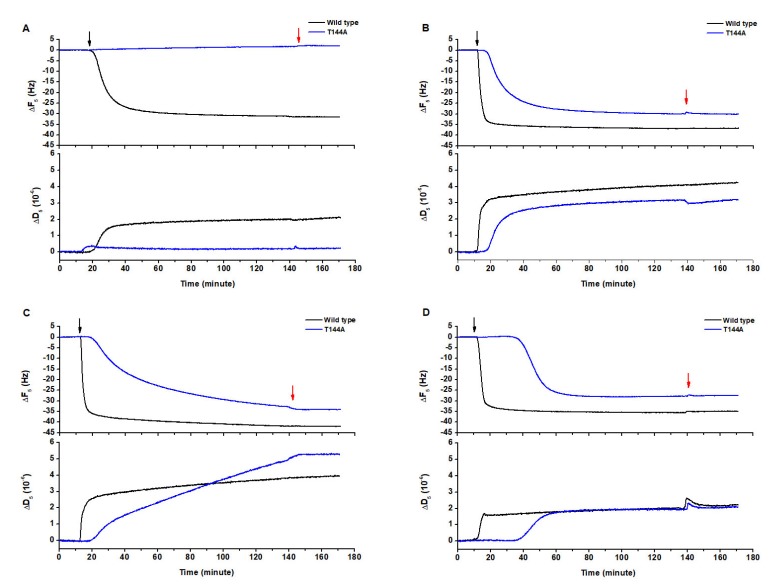
Protein-lipid binding of Cyt2Aa2 wild type and the T144A mutant on different lipid bilayers. The lipid bilayers were formed on the surface of silica sensors. Once the bilayer was built, the value of the frequency was set to zero. Thus, the reported difference in frequency relates to the adsorption of the protein toxin on the lipid bilayers. The protein solution (25 µg/mL) was filled into the quartz crystal microbalance with dissipation (QCM-D) chamber, and then the flow was paused in order to evaluate the Cyt2Aa2-lipid binding for 2 h. The black arrow and red arrow indicate protein exposure and buffer rinsing, respectively. (**A**) phospholipid (POPC), (**B**) 1:0.4 POPC/cholesterol (Chol), (**C**) 1:1 sphingomyelin (SM)/POPC and (**D**) 1:1:1 SM/POPC/Chol.

The lipid components of the cell membrane, phospholipid (POPC), sphingomyelin (SM) and cholesterol (Chol), were mixed in different molar ratios in order to form different lipid bilayers that could mimic the erythrocyte cell membrane. The interaction of both Cyt2Aa2 wild type (WT) and T144A mutant with pure POPC (Figure 1A), 1:0.4 POPC/Chol (Figure 1B), 1:1 SM/POPC (Figure 1C), and 1:1:1 SM/POPC/Chol bilayers was investigated with QCM-D (Figure 1D). The results showed that Cyt2Aa2 WT (black plot) interacted with all kind of lipid bilayers, whereas no interaction between the T144A mutant and the POPC bilayer could be detected. In this case, the frequency (ΔF) and the dissipation (ΔD) signals did not change with time (blue plot). The measurements indicated that the lipid binding of both the Cyt2Aa2 WT and T144A mutant was saturated for ΔF values between −25 to −40 Hz. The difference in dissipation (ΔD) achieved values between 2 × 10^−6^ to 5 × 10^−6^ (Table 1). Here it is worth remembering that ΔF relates to changes in the mass adsorption (on the sensor surface), and that ΔD refers to the viscoelastic properties of the formed hybrid protein-lipid layer.

**Figure 2:**
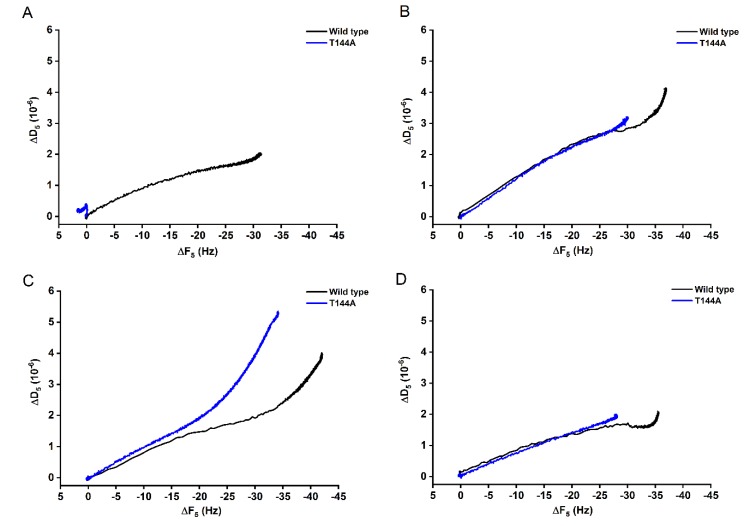
ΔD-ΔF plots of the binding of Cyt2Aa2 WT (black) and the T144A mutant (blue) on different model lipid bilayers. The dissipation value (ΔD) was plotted against the frequency value (ΔF) to elucidate the interplay between the protein binding and the viscoelasticity of the hybrid protein-lipid layer. The similarity of the slopes indicates an analogous qualitative behavior. (**A**) POPC, (**B**) 1:0.4 POPC/Chol, (**C**) 1:1 SM/POPC, and (**D**) 1:1:1 SM/POPC/Chol bilayers.

In addition, ΔD-ΔF plots can be used to compare the binding behavior between the WT and the mutant T144A. For binding onto POPC bilayers (Figure 2A), the ΔD-ΔF signal for the T144A remained mostly constant with increasing time, while the WT showed a proportional increasing of ΔD and ΔF. However, the WT and the T144A seemed to bind in a similar way onto 1:0.4 POPC/Chol and 1:1:1 SM/POPC/Chol bilayers (Figure 2B,D), suggesting similar viscoelastic properties. On the contrary, a different trend occurred for the binding onto 1:1 SM/POPC bilayers (Figure 2C). It can be observed that for the same frequency change (ΔF) the mutant induced a final less rigid protein-lipid layer.

Furthermore, the binding kinetics were determined by fitting the experimental data to a single exponential decay equation:

(1) $$F_{t}{=F}_{0}{+Ae}^{-t/\Gamma }$$

(see Figure S2). In Table 1, the constant decay (*Γ*) indicates the lipid binding rate. Thus, a lower value means a faster binding rate, and vice versa. It can be observed that the WT showed lower *Γ* values than the T144A mutant for all types of model lipid bilayers. Hence, the binding rate of the WT was faster than the T144A mutant. Remarkably, the *Γ* values of the WT corresponding to the 1:0.4 POPC/Chol, 1:1 SM/POPC and 1:1:1 SM/POPC/Chol bilayers (ca. 2.0 min) were approximately five times smaller than the rate for the POPC bilayers (ca. 10.0 min). It seemed that the lipid bilayers containing either cholesterol or sphingomyelin favored the binding of Cyt2Aa2 WT. Similarly, the binding of the T144A mutant could be detected when cholesterol or sphingomyelin were present in the lipid bilayers. However, the *Γ* values indicate that the lipid binding ability of T144A mutant onto 1:1 SM/POPC bilayers (*Γ* = 52.1 min) was lower than 1:0.4 POPC/Chol (*Γ* = 11.2 min) and 1:1:1 SM/POPC/Chol (*Γ* = 10.9 min) bilayers, respectively. Unlike the WT case, sphingomyelin promoted a lower binding capability of the T144A mutant than cholesterol. These findings indicate that the replacement of threonine 144 with alanine results in a reduction of the lipid binding ability of Cyt2Aa2 toxin, especially for POPC bilayers.

### 2.2. AFM Imaging of the Cyt2Aa2 (WT and Mutant) Interaction with Different Model Lipid Bilayers

**Figure 3:**
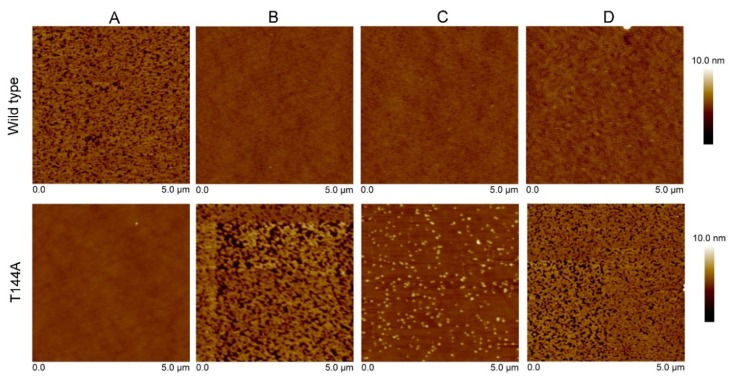
Atomic force microscopy (AFM) height images showing the interaction of the Cyt2Aa2 WT and the T144A mutant with the different lipid bilayers. First, the lipid bilayers were formed on silica surfaces. After, both protein solutions (WT and mutant) were exposed to the lipid bilayers for 30 min. The AFM images were collected in tapping mode with a scan rate of 1–2 Hz. Note that the scan size of every image is 5 µm × 5 µm. The vertical scale (until 10 nm) is indicated on the right. Image processing was carried out with the Nanoscope program. (**A**) POPC, (**B**) 1:0.4 POPC/Chol, (**C**) 1:1 SM/POPC, and (**D**) 1:1:1 SM/POPC/Chol bilayers.

AFM experiments were carried out in order to investigate the topographic structure of the different Cyt2Aa2-lipid layers. The model lipid bilayers were successfully formed on the silica surface via lipid vesicle fusion and revealed a smooth surface (Figure S3). Subsequently, the protein solutions with the Cyt2Aa2 WT and the T144A mutant were incubated with the lipid bilayers. The surface topography of the hybrid protein-lipid layers was visualized after 30 min of incubation. Figure 3 shows no binding of the T144A mutant on POPC bilayers, which agrees with the QCM-D results. In contrast, the WT toxin almost covered the whole lipid bilayer surface (black areas refer to protein-free lipid bilayer). A longer incubation time of 120 min did not promote the binding of the T144A mutant on the POPC bilayer (Figure S4A). Subsequently, cholesterol and sphingomyelin were included into the lipid mixtures. Cyt2Aa2 WT bound onto the lipid surfaces reaching saturation. Thus, the lipid surfaces were fully covered with Cyt2Aa2 WT (note that the black areas disappeared). In addition, the binding between the T144A mutant and the lipid bilayers could be observed. The binding behavior of the T144A mutant onto 1:0.4 POPC/Chol and 1:1:1 SM/POPC/Chol bilayers showed a similar trend than the trend depicted by the WT; the protein-free membrane (black area) was observed prior to reaching a saturation after 120 min (see Figure S4). Remarkably, the T144A mutant formed small protein aggregates onto 1:1 SM/POPC bilayers. These aggregates seemed to be different from the ones observed for the WT and the T144A mutant on other lipid membranes (Figure 3).

**Figure 4:**
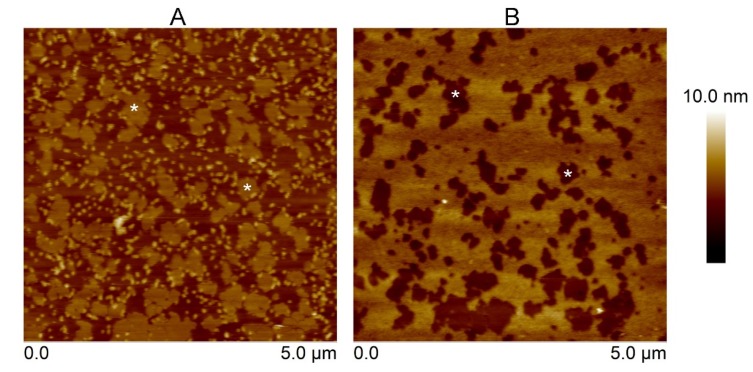
AFM height images of the Cyt2Aa2 wild type and the T144A mutant on 1:1 SM/DOPC (1,2-dioleoyl-*sn*-glycero-3-phosphocholine) bilayers. The SM domains are indicated as white asterisks. The lipid bilayers were initially exposed to the T144A mutant (25 µg/mL) for 2 h (**A**). After buffer rinsing, the Cyt2Aa2 wild type solution (25 µg/mL) was exposed to the lipid bilayers for 1 h (**B**). The topographic images were collected in tapping mode at a scan rate of 1–2 Hz. Note that the scan size of both images is 5 µm × 5 µm. The vertical scale (until 10 nm) is indicated on the right. Image analysis was performed with the Nanoscope program.

Furthermore, the influence of sphingomyelin (SM) on the binding capability of the Cyt2Aa2 toxins was determined. For this purpose, 1:1 SM/DOPC (1,2-dioleoyl-*sn*-glycero-3-phosphocholine) bilayers were exposed to the toxins. For the SM/DOPC bilayers, a phase separation was observed where the sphingomyelin domains appeared as a liquid disordered-solid phase (l_d_-S_o_). In particular, the S_o_ domains of SM were distributed over the lipid bilayer surface being about 1 nm thicker than the DOPC-enriched domains (Figure S5). The 1:1 SM/DOPC bilayers were firstly exposed to the T144A mutant. The observed protein aggregates looked similar to the aggregates found on the SM/POPC bilayers. Subsequently, the WT protein was introduced into the system. The AFM micrographs indicate that the WT fully occupied the remaining areas (DOPC-enriched domains). Furthermore, no protein could be observed on SM domains (Figure 4). This suggests unfavorable binding of the Cyt2Aa2 toxins onto SM bilayers. Concordantly, AFM and QCM-D results support each other (i.e., no binding of the T144A mutant on the POPC bilayer). Moreover, AFM topography studies provided additional information about the T144A-lipid complex formation onto SM/POPC bilayers and its binding inability onto sphingomyelin bilayers.

### 2.3. Cyt2Aa2 (WT and Mutant) Toxin Interaction with Erythrocyte Cell Membranes

Sheep erythrocytes presented a round and concave shape with diameter of ~3.0 µm under the light microscope. To prepare the erythrocyte membrane layers, a low salt solution (1/3 dilution PBS) was used to break the cell attached on the supporter surface. The ghost erythrocytes appeared as flat cells because of the releasing of cytoplasmic fluid (Figure S6). AFM images revealed a size of ca. 3.0–4.0 µm for the erythrocytes, which agreed with light microscopy observations.

**Figure 5:**
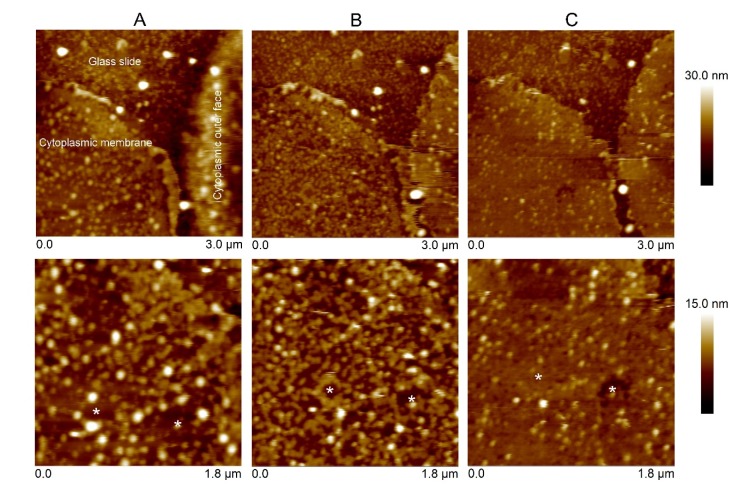
AFM images of Cyt2Aa2 toxin binding on the erythrocyte membrane. The erythrocyte membrane was prepared on a lysine-coated glass (**A**). The cell membranes were initially exposed to the T144A mutant (25 µg/mL) for 2 h (**B**). After buffer rinsing, the Cyt2Aa2 wild type solution (25 µg/mL) was exposed to the same membrane for 1 h (**C**). The topographic images were collected in tapping mode at a scan rate of 1–2 Hz. The images were analyzed with the Nanoscope program. The images have a scan size of 3 µm × 3 µm (upper panel) and 1.8 µm × 1.8 µm (lower panel). Note that the vertical scale differs: from 0 to 30 nm (upper panel), and from 0 to 15 nm (lower panel). The white asterisks mark the same areas on the erythrocyte membrane.

After the erythrocytes were lysed, it was assumed that two types of erythrocyte membrane could be observed: (i) a single layer (inner cytoplasmic membrane) formed by cell opening (inside membrane facing up), and (ii) a double layer presenting the outer surface of the membrane (inner and outer cytoplasmic membranes) (Figure 5). Unlike the model lipid bilayers, the erythrocyte cytoplasmic membrane had a rougher surface. The membranes were firstly exposed to the T144A mutant. The binding of the T144A revealed a change in the topography of the height surface (area surrounding the asterisks). However, some areas of the membrane remained free of the T144A protein (no binding). In a second step, Cyt2Aa2 WT was introduced into the system. Cyt2Aa2 WT bound to the remaining areas leading to a smoother surface (compared to the erythrocyte-T144A ones) (Figure 5). The experiments with model lipid bilayers showed that the T144A mutant could not bind to the POPC bilayer bilayers. Therefore, it was not expected that the mutant would bind on the POPC areas of the erythrocyte membrane. On the contrary, the results of Cyt2Aa2 WT could indicate that WT bind POPC-enriched domains of the erythrocyte membrane.

## 3. Discussion

In this work, we have studied the interaction of two Cyt2Aa2 proteins, the WT and the less hemolytic T144A mutant [24], with model lipid bilayers and erythrocyte membranes. QCM-D results showed that the T144A mutant could not bind to the POPC bilayer. In turn, the mutant protein retained its binding capability when exposed to 1:0.4 POPC/Chol, 1:1 SM/POPC, and 1:1:1 SM/POPC/Chol bilayers (Figure 1). Moreover, the ΔD–ΔF plots suggest that the binding behavior of the WT and T144A mutant followed similar adsorption trends except for 1:1 SM/POPC bilayers (Figure 2). The final values of ΔF and ΔD were not significantly different for the Cyt2Aa2 WT and the T144A mutant (Table 1). Our values are similar to the reported values for the binding of perforin on lipid membranes [27]. However, the mutant presented a lipid binding rate of at least five times lower than the WT, suggesting a smaller binding ability of the T144A mutant. The presence of either cholesterol or sphingomyelin in the lipid bilayer increased the binding rate of the WT and promoted the binding of the T144A mutant on the model lipid bilayers. At room temperature (25 °C), the POPC bilayer exists in a liquid disordered phase (l_d_). Addition of cholesterol or sphingomyelin into lipid membranes reduces the fluidity of the lipid bilayer (less lateral diffusion) [28]. Less dynamic membranes seem to increase the possibility for the Cyt2Aa2-lipid interaction. Hence, Cyt2Aa2 WT bound faster on the 1:0.4 POPC/Chol, 1:1 SM/POPC and 1:1:1 SM/POPC/Chol bilayers than on the single POPC bilayer. In addition, it was observed that the T144A mutant could also bind onto these heterogeneous membranes, but not onto POPC bilayers. It seems that although the membranes of insect cells and mammalian cells are different in composition, both support the larvicidal activity and the low hemolytic activity of the T144A mutant. In comparison to mammalian cells, insect cell membranes present a lower amount of cholesterol and sphingomyelin [29]. This might suggest that the binding of the Cyt2Aa2 toxin on insect cells is possibly promoted by other components of the cell membrane (e.g., membrane proteins) besides cholesterol and sphingomyelin. This different binding mechanism might play a role in the in vivo larvicial activity of the Cyt2Aa2 toxin.

Atomic force microscopy (AFM) was carried out to investigate the surface structure of the hybrid Cyt2Aa2-lipid bilayers. The AFM measurements confirmed that the T144A mutant does not bind onto POPC bilayers, while the WT toxin adsorbs on such lipid bilayers (Figure 3). With the addition of cholesterol into this lipid bilayer, the 1:0.4 POPC/Chol bilayer led to the binding of the T144A mutant. On the contrary, sphingomyelin (1:1 SM/POPC bilayer) also promoted the T144A mutant binding but a dissimilar binding was observed as well as small protein aggregates. Correspondingly, the ΔD–ΔF plots of QCM-D results suggest dissimilar binding behavior between the WT and the T144A mutant on the 1:1 SM/POPC bilayers (Figure 2). Furthermore, the binding behavior of Cyt2Aa2 WT and the T144A mutant were very much alike once cholesterol was included into the lipid membranes, as found for the 1:0.4 POPC/Chol and 1:1:1 SM/POPC/Chol bilayers (Figure 3). Moreover, both Cyt2Aa2 WT and the T144A mutant did not bind onto sphingomyelin (S_o_) domains (Figure 4). These results suggest that although the lipid head group plays a role in the interaction between Cyt2Aa2 toxin and lipid bilayers, the lipid phase might also be taken into account [30,31]. This could be feasible since sphingomyelin contains the same choline head group as POPC. Besides, the small aggregates of the T144A mutant might imply a coexistence of a different fluid membrane in the 1:1 SM/POPC bilayer. This particular result indicates that cholesterol is more important for the binding behavior of the T144A mutant than for the binding of Cyt2Aa2 WT.

Furthermore, the interaction of Cyt2Aa2-lipid (WT and mutant) with sheep erythrocyte membranes was investigated. Unlike model lipid bilayers, a rougher surface was detected for the sheep erythrocyte membrane, which is comparable to the chicken erythrocyte membrane [32], and the human erythrocyte membrane [33]. The T144A mutant revealed a limited binding capability on the erythrocyte membrane, leaving part of the membrane uncovered. Further experiments indicated that the Cyt2Aa2 WT could bind on the remaining free areas (Figure 5). This binding study provides insight about the different properties of the erythrocyte membranes, e.g., lipid composition and lipid fluidity, when compared with model lipid membranes. Nowadays, a detection of the lipid phase coexistence in biological cell membranes is still a challenge for a biologist. Although lipid phase separation could not be observed in this experiment, the two Cyt2Aa2 proteins enabled us to distinguish the different components of the lipid membrane of sheep erythrocytes.

In conclusion, the combination of QCM-D and AFM permitted us to monitor the interaction between CytAa2 toxin with model lipid bilayers and supported erythrocyte membranes. The lower protein-lipid binding capability of the T144A mutant (in comparison with the WT) could lead to its small hemolytic activity. In particular, the alanine replacement of threonine 144 residue disables the binding properties of the Cyt2Aa2 toxin onto the POPC bilayer. Although certain hemolytic activity still remains for the T144A mutant, it can be said that the T144 residue located in the αD-β4 loop plays an important role in the Cyt2Aa2-lipid binding. Furthermore, the modification of amino acid residues in the αD-β4 loop of the Cyt2Aa2 toxin will be investigated for specific cell targeting. In future work, the effect of different amino acid properties (e.g., polar charge and positive charge) on the Cyt2Aa2-lipid interaction will be investigated. In addition, protein concentration, lipid phase and lipid charge will be taken into account for further investigations.

## 4. Materials and Methods

### 4.1. Reagents and Buffer

1-palmitoyl,2-oleoyl-sn-glycero-3-phosphocholine (POPC), 1,2-dioleoyl-*sn*-glycero-3-phosphocholine (DOPC), chicken egg yolk sphingomyelin (SM), and cholesterol (Chol) were purchased from Sigma-Aldrich (Darmstadt, Germany). The lipids were dissolved in chloroform and divided into 1 mg aliquots. Then, the organic solvent was evaporated under nitrogen stream and kept at −20 °C.

Phosphate buffered saline (PBS) pH 7.4 (137 mM NaCl, 2.7 mM KCl and 10 mM phosphate) was prepared from PBS tablet (Sigma-Aldrich, Darmstadt, Germany). The buffer tablet was dissolved in ultrapure water (Milli-Q, Merck, Darmstadt, Germany) and filtrated through a 0.22 µm filter (Whatman, GE Health care life science, Chicago, IL, USA).

### 4.2. Protein Preparation

The Cyt2Aa2 toxin from *Bacillus thuringiensis* subs. *darmstadiensis* was expressed in *Escherichia coli* as previously described by B. Promdonkoy [14]. The amino acid replacement at the threonine 144 residue with alanine was carried out by means of site-directed mutagenesis as described in a previous publication [24]. To obtain activated Cyt2Aa2 toxin (25 kDa), the Cyt2Aa2 inclusion was solubilized in 50 mM carbonate buffer, pH 10.0 at 30 °C for 1 h. The soluble Cyt2Aa2 toxin (29 kDa-protoxin) was collected by centrifugation at 10,000× *g* for 10 min. Then, the Cyt2Aa2 toxin was activated by 2% (*w*/*w*) chymotrypsin (Sigma-Aldrich, Darmstadt, Germany) at 30 °C for 2 h. The purity of the protein was determined by SDS-PAGE (Invitrogen, Waltham, MA, USA). Protein concentration was determined by UV adsorption (Hitachi, Tokyo, Japan). Stock protein solution was prepared to 2.0 mg/mL (80 µM) and kept at −20 °C.

### 4.3. Lipid Vesicle Preparation

The lipids were mixed in chloroform with the desired lipid ratios. After that, the organic solvent was evaporated under a gentle nitrogen stream to form lipid films. The residual solvent was removed by further keeping the lipid films under nitrogen stream for 1 h. Furthermore, the lipid films were hydrated with PBS solution to a concentration of 1 mg/mL and incubated above the melting transition temperature (*T_m_*) for 2 h. The hydrated films were intermittently vortexed during incubation until complete suspension. The vesicles were homogenized by extrusion method for low *T_m_* lipid mixtures (POPC/Chol system). The vesicles were pressed through a 50 nm Øpolycarbonate membrane for 21 times at room temperature by using a mini-extruder (Avanti, Alabaster, AL, USA). For the lipid mixtures with higher *T_m_* (SM system) tip sonication with a 50% duty cycle of 10 min was used (Branson sonifier, Emerson, Ferguson, MO, USA). Then, the residual material was removed by centrifugation at 10,000× *g* for 10 min. After that, the vesicles size, in a range of 100–130 nm, was determined by Zetasizer Nano ZS (Malvern Instrument, Worcestershire, UK). The vesicle solutions were stored at a temperature higher than *T_m_* and were used within a week.

### 4.4. Supported Erythrocyte Cell Membrane Preparation

Sheep blood (Oxoid, Thermo scientific, Waltham, MA, USA) was removed by washing with PBS pH 7.4 three times. The sheep blood was gentle mixed with PBS in a ratio of 1:7. Then, the erythrocytes were collected by centrifugation at 3000× *g* and 4 °C for 5 min. The erythrocyte pellet was kept and resuspended in PBS pH 7.4, 2% (*V*/*V*), as a working solution.

The erythrocyte membrane was prepared on a poly-lysine coated glass. The round-glass cover slips were cleaned as follows: soaking in 1.0 M hydrochloric acid for 2 h, rinsing thoroughly with ultrapure water (MilliQ, Merck, Darmstadt, Germany), sonication in 70% (*v*/*v*) ethanol for 10 min, and final treatment with plasma cleaner (Diener electronic, Ebhausen, Germany). Prior cell attachment, the glass cover slips were coated with 30–70 kDa poly L-lysine (Sigma, Darmstadt, Germany). The glass slips were immersed in a 0.1 mg/mL lysine solution (in PBS) for 30 min at room temperature. The excess of lysine was removed by buffer rinsing. After that, the erythrocytes were attached on the glass surface by incubation over the surface for 30 min at room temperature. The unbound erythrocytes were removed and the attached cells were opened under shear flow by using a low content salt solution (1/3 dilution PBS; 45.7 mM NaCl, 0.9 mM KCl and 3.3 mM phosphate). Finally, the cell membrane was rinsed with PBS pH 7.4.

### 4.5. Quartz Crystal Microbalance with Dissipation (QCM-D) Measurement

The protein-lipid bilayer interaction was evaluated with quartz crystal microbalance with dissipation from Q-Sense E4 (Biolin Scientific, Gothenburg, Sweden) using silica-coated sensors (QSX 303, Biolin Scientific, Sweden). Before use, the sensors were subsequently cleaned as follows: sonication in 2% (*w*/*w*) SDS solution for 15 min, rinsing with ultra-pure water, drying under nitrogen stream, and organic residues-eliminating with UV/Ozone cleaner (Bioforce Nanosciences, Salt Lake City, UT, USA) for 30 min. The frequencies of the sensors were evaluated prior to running the experiments. The outcome of the experiments delivers changes in frequency and dissipation. The change in frequency (Δ*F*) is proportional to changes in the adsorbed mass (Δm) on the crystal surface through the Sauerbrey equation:

(2) Δm=−CnΔF

where (*C*) is the sensitivity constant (−17.7 ng cm^−2^ Hz^−1^) and (*n*) is the overtone number. Simultaneously, the change in dissipation (Δ*D*) indicates the viscoelastic properties of the new forming layer on the crystal surface (in our case, of the hybrid protein-bilayer system). Low dissipation values are typical for a rigid (elastic) layer whereas high values relate to softer (viscoelastic) layers. The changes in frequency (Δ*F*) and dissipation (Δ*D*) values are presented for the 5th overtone unless otherwise stated.

The lipid bilayers were formed by the lipid vesicle fusion method. After a stable baseline with PBS solution was achieved, 0.1 mg/mL lipid vesicle solutions were slowly flowed in the QCM-D chamber with a flow rate of 50 µl/min. Once the characteristic patterns (for the frequency and dissipation) of lipid bilayer formation were observed, the excess of vesicles was removed by buffer rinsing. Some of the lipid bilayers were completely formed by additional water rinsing (through osmotic stress). Finally, all lipid bilayers were incubated under PBS flow until reaching a stable baseline.

To study the interaction of the toxin with the model lipid bilayers, the different Cyt2Aa2 toxin solutions were introduced into the system at a flow rate of 50 µL/min. After that, the flow was stopped in order to evaluate when the protein-lipid binding could reach a saturated state. Furthermore, the unbound protein was flushed from the chamber with PBS solution at a flow rate of 50 µL/min for 30 min. The experiments were carried out with at least three replications at 25 °C (298 K). The protein-lipid binding kinetics was determined by curve fitting. The frequency (ΔF) vs. time plots were fitted with a single exponential decay equation (Equation (1)). All the plots and data fitting were carried out with Origin 8.0 (OriginLab Corporation, Northampton, MA, USA).

### 4.6. Atomic Force Microscopy (AFM) Imaging

The AFM cantilevers with a nominal spring constant of 0.24 N/m (DNP-S10, Bruker, Billerica, MA, USA) and the silica substrate were mounted inside a closed fluid cell with an O-ring. The 1 cm × 1 cm silica wafers (IMEC, Leuven, Belgium) were cleaned before using with the following procedure: sonication in 2% (*w*/*w*) SDS solution for 15 min, rinsing with ultrapure water, and drying under nitrogen stream. Finally, the substrates were treated by plasma cleaner (Diener electronic, Ebhausen, Germany). The lipid bilayers were formed by means of lipid vesicle fusion. 0.1 mg/mL of lipid vesicle solutions were incubated over the silica surface for at least 10 min and then the vesicle excess was rinsed from the chamber. Afterwards, the two Cyt2Aa2 proteins, wild type (WT) and the T144A mutant (25 µg/mL or 1.0 µM), were incubated with the lipid bilayers or with supported erythrocyte membrane for the desired experimental time. The surface topography was imaged in tapping mode with a JV-scanner controlled by a NanoScope V controller (Bruker, Billerica, MA, USA) at a scan rate of 1–2 Hz. The images were processed and analyzed with the Nanoscope program. The experiments were carried out at room temperature (298 K).

## Figures and Tables

**Table 1:** ΔF, ΔD, and lipid binding rate values for wild type (WT) and T144A on different lipid bilayers.

Lipid Composition (Mole Ratio)	ΔF_5_ (Hz)		ΔD_5_ (10^−6^)		Lipid Binding Rate, Γ (min)	
	WT	T144A	WT	T144A	WT	T144A
POPC	−33.0 ± 3.8	1.3 ± 0.4	2.9 ± 1.1	0.0 ± 0.2	9.5 ± 0.3	No binding
1:0.4 POPC/Chol	−38.0 ± 1.7	−29.1 ± 0.6	3.1 ± 1.0	2.3 ± 0.7	2.1 ± 0.2	11.2 ± 2.0
1:1 SM/POPC	−40.1 ± 3.4	−24.4 ± 10.6	5.0 ± 0.2	3.0 ± 2.2	2.2 ± 0.6	52.1 ± 27.3
1:1:1 SM/POPC/Chol	−30.2 ± 4.4	−25.4 ± 2.4	2.3 ± 2.0	2.2 ± 0.2	2.5 ± 0.4	10.9 ± 1.9

## Supplementary Materials

The following are available online at https://www.mdpi.com/2072-6651/12/4/226/s1, Figure S1: Three dimensional structure of the Cyt2A toxin (PDB 1CBY). Figure S2: Curve fitting of the frequency vs. time plots of the binding between the Cyt2Aa2 toxins and the model lipid bilayers. Figure S3: AFM images of the lipid bilayers. Figure S4: Time sequence AFM imaging of the binding of the Cyt2Aa2 toxins on lipid bilayers. Figure S5: AFM height images and profile analysis of hybrid Cyt2Aa2-1:1 SM/DOPC bilayers. Figure S6: Cell shape of sheep erythrocytes under light microscopy.
