# Supplementary material for: Protein-Lipid Interaction of Cytolytic Toxin Cyt2Aa2 on Model Lipid Bilayers of Erythrocyte Cell Membrane
Source: Toxins (Basel). 2020 Apr 3;12(4):226. doi: 10.3390/toxins12040226 (PMC7232533; doi:10.3390/toxins12040226)
Supplement: Supplementary file 1 [file toxins-12-00226-s001.pdf]

Supplementary Materials: Protein-Lipid Interaction of Cytolytic Toxin Cyt2Aa2 on Model Lipid Bilayers of Erythrocyte Cell Membrane

Sudarat Tharad *, Boonhiang Promdonkoy and José L. Toca-Herrera *

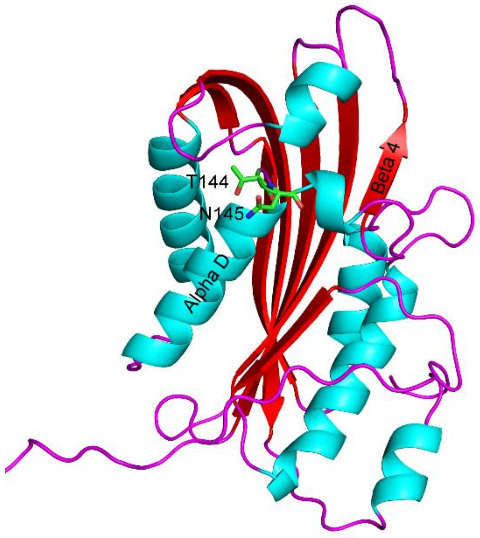

Figure S1. Three dimensional structure of the Cyt2A toxin (PDB 1CBY). Note the location of the T144 residue in the αD–β4 loop.

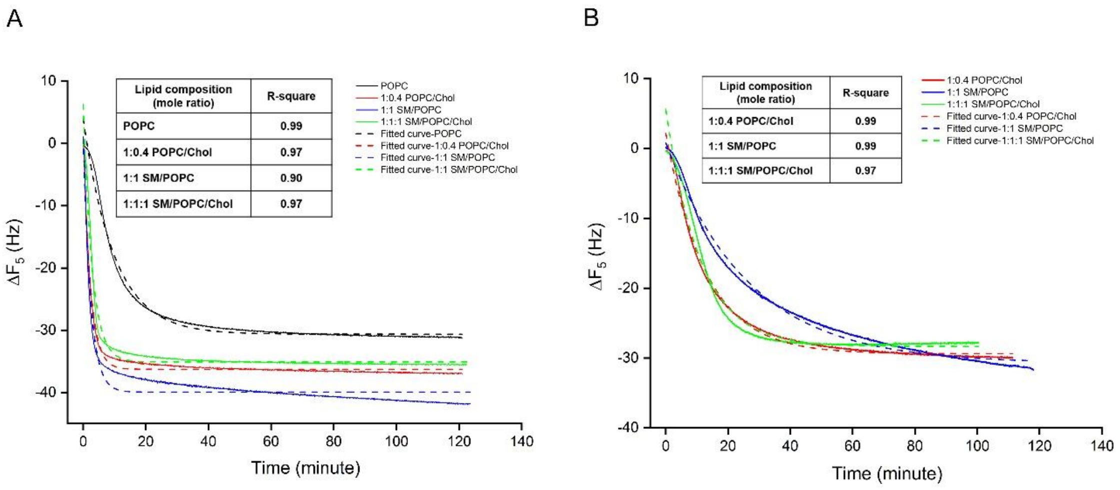

Figure S2. Curve fitting of the frequency vs. time plots of the binding between the Cyt2Aa2 toxins and the model lipid bilayers. The frequency plots (ΔF) were fitted with a single exponential decay equation: $F_t = F_0 + Ae^{-t/\Gamma}$ in order to determine the binding rates. The time constant of decay (Γ) refers to the protein binding rate on the lipid bilayers. (**A**) Cyt2Aa2 wild type and (**B**) Cyt2Aa2 T144A mutant.

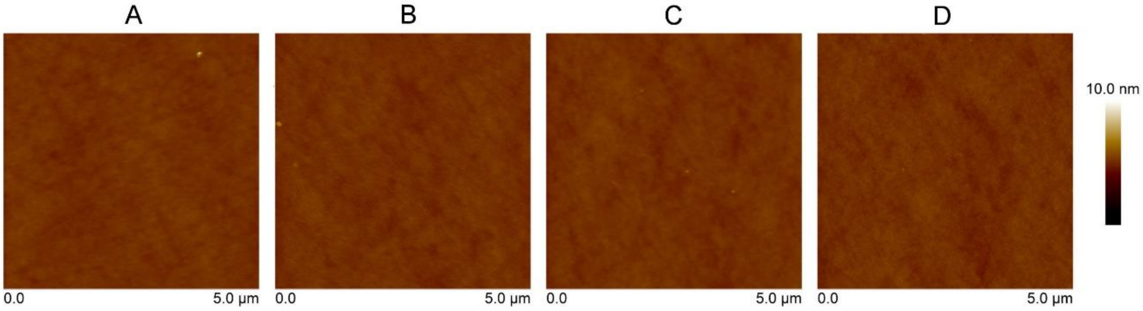

Figure S3. AFM images of the lipid bilayers. The lipid bilayers were formed on silica surfaces via lipid vesicle fusion. The lipid vesicles were incubated over the surface for at least 10 min. After that time the remaining lipid vesicles were removed from the system. The surfaces were visualized by AFM tapping mode with a scanning rate of 1–2 Hz. Note that the scan size of every image is 5 µm × 5 µm. (**A**) POPC, (**B**) 1:0.4 POPC/Chol, (**C**) 1:1 SM/POPC, and (**D**) 1:1:1 SM/POPC/Chol bilayers.

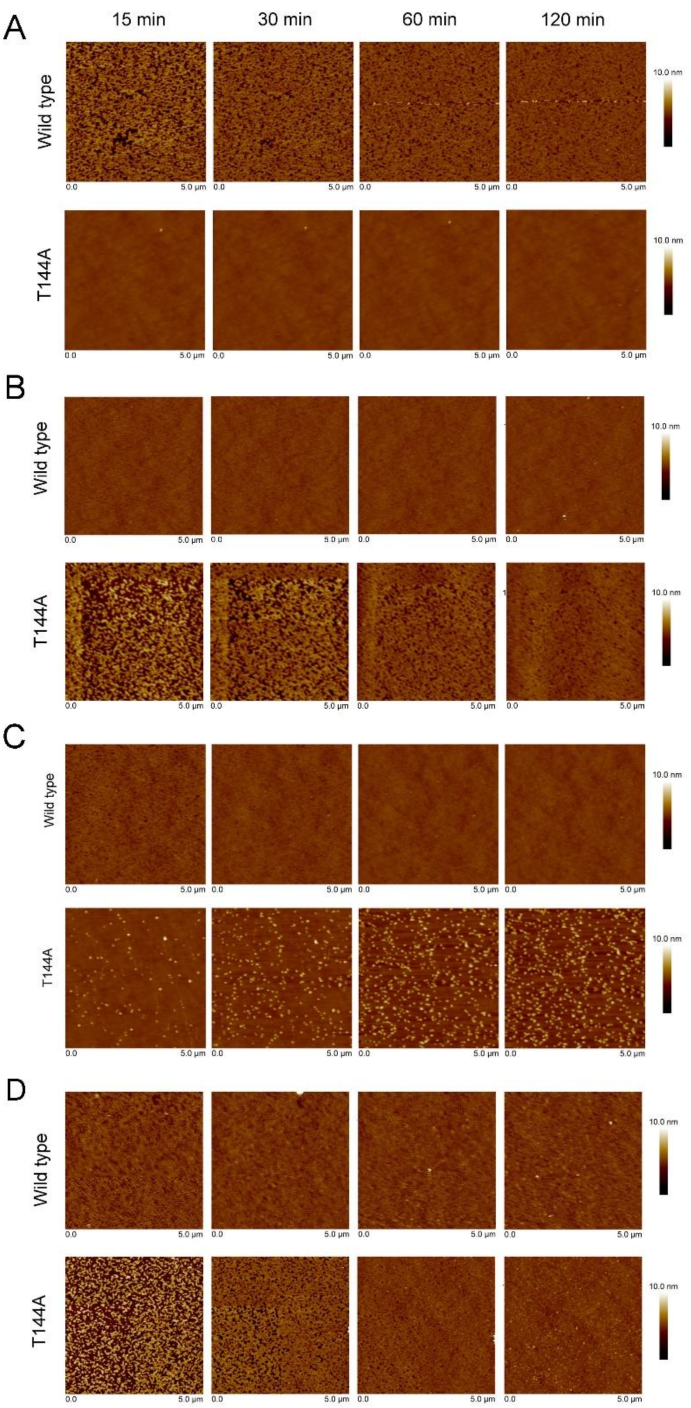

Figure S4. Time sequence AFM imaging of the binding of the Cyt2Aa2 toxins on lipid bilayers. Both Cyt2Aa2 wild type and T144A mutant (25 µg/mL) were exposed to the lipid bilayers and incubated for 2 h. The AFM images were collected as a function of time in tapping mode with a scan rate of 1–2 Hz. Note that the scan size of every image is 5 µm × 5 µm. The images were processed by Nanoscope program. (**A**) POPC, (**B**) 1:0.4 POPC/Chol, (**C**) 1:1 SM/POPC and (**D**) 1:1:1 SM/POPC/Chol.

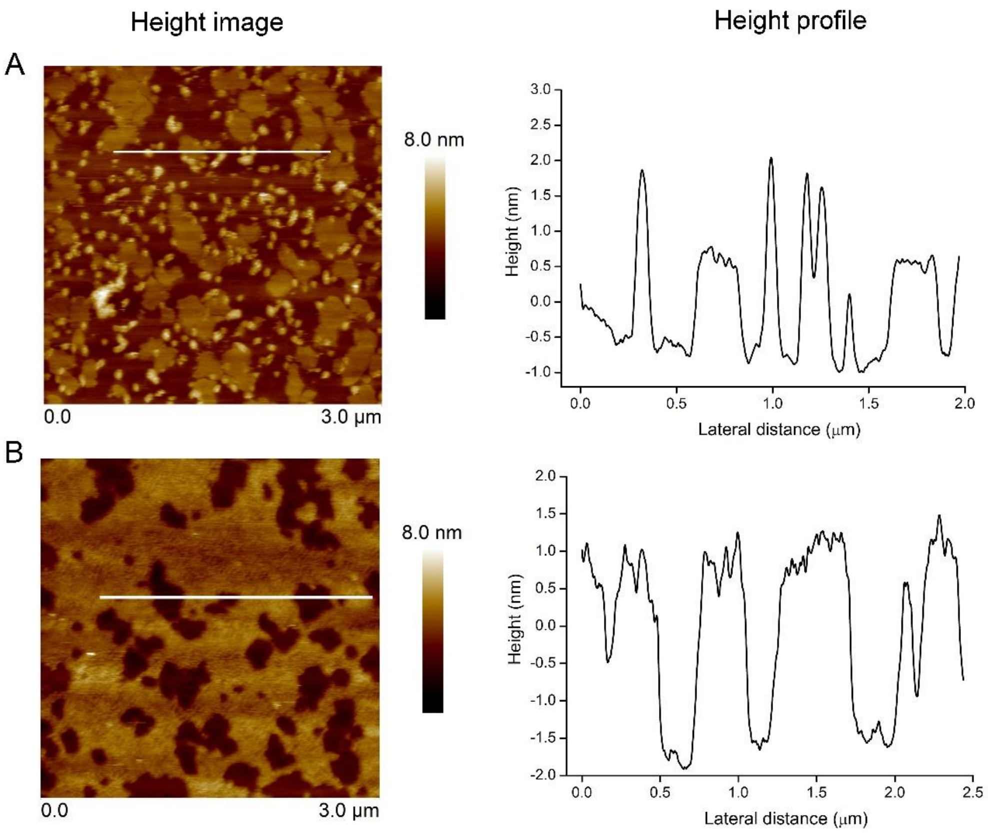

Figure S5. AFM height images and profile analysis of hybrid Cyt2Aa2-1:1 SM/DOPC bilayers. The height images show the topography changes induced by the interaction of the Cyt2Aa2 T144A mutant (**A**) and Cyt2Aa2 wild type (**B**) with 1:1 SM/DOPC bilayers (left panel). The height profiles (right panel) correspond to the white line of drawn on the height images. Note that the difference in height (thickness) varies between 1 nm and 3 nm. The scan size of both images is 3 µm × 3 µm.

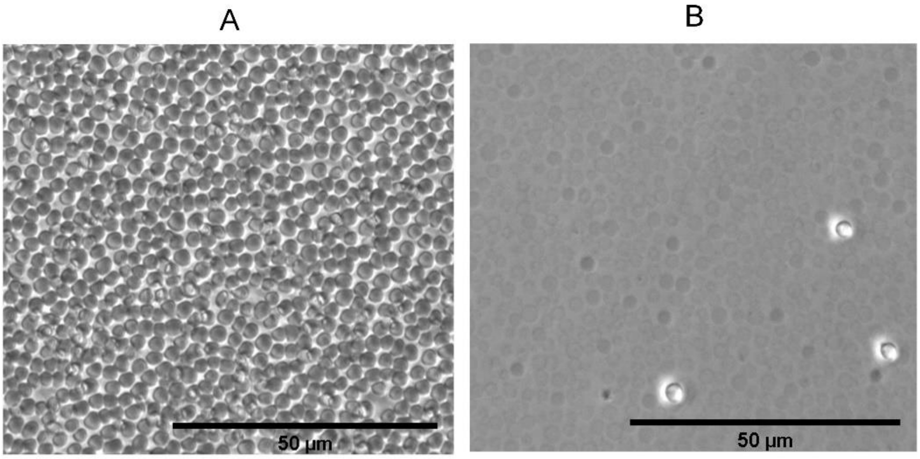

Figure S6. Cell shape of sheep erythrocyte under light microscopy. Sheep erythrocytes (in PBS pH 7.4) were deposited on lysine-coated glass slide and visualized with a light microscope (Nikon Eclipse TE2000-S, Nikon, Vienna, Austria). Concave shapes were observed for the erythrocytes in PBS (**A**) while the flat shapes were seen for the erythrocytes in the low salt solution (1/3 dilution PBS) (**B**). Scale bar is 50 µm.
